# Prediction of mammalian tissue-specific CLOCK–BMAL1 binding to E-box DNA motifs

**DOI:** 10.1038/s41598-023-34115-w

**Published:** 2023-05-12

**Authors:** Daniel Marri, David Filipovic, Omar Kana, Shelley Tischkau, Sudin Bhattacharya

**Affiliations:** 1 Department of Biomedical Engineering, Michigan State University, East Lansing, MI USA; 2 Institute for Quantitative Health Science and Engineering, Michigan State University, East Lansing, MI USA; 3 Department of Computational Mathematics, Science and Engineering, Michigan State University, East Lansing, MI USA; 4 Department of Pharmacology and Toxicology, Michigan State University, East Lansing, MI USA; 5 Institute for Integrative Toxicology, Michigan State University, East Lansing, MI USA; 6 Department of Pharmacology, Southern Illinois University School of Medicine, Springfield, IL USA

**Keywords:** Computational biology and bioinformatics, Systems biology

## Abstract

The Brain and Muscle ARNTL-Like 1 protein (BMAL1) forms a heterodimer with either Circadian Locomotor Output Cycles Kaput (CLOCK) or Neuronal PAS domain protein 2 (NPAS2) to act as a master regulator of the mammalian circadian clock gene network. The dimer binds to E-box gene regulatory elements on DNA, activating downstream transcription of clock genes. Identification of transcription factor binding sites and genomic features that correlate to DNA binding by BMAL1 is a challenging problem, given that CLOCK–BMAL1 or NPAS2–BMAL1 bind to several distinct binding motifs (CANNTG) on DNA. Using three different types of tissue-specific machine learning models with features based on (1) DNA sequence, (2) DNA sequence plus DNA shape, and (3) DNA sequence and shape plus histone modifications, we developed an interpretable predictive model of genome-wide BMAL1 binding to E-box motifs and dissected the mechanisms underlying BMAL1–DNA binding. Our results indicated that histone modifications, the local shape of the DNA, and the flanking sequence of the E-box motif are sufficient predictive features for BMAL1–DNA binding. Our models also provide mechanistic insights into tissue specificity of DNA binding by BMAL1.

## Introduction

All animals and plants have a robust time-keeping mechanism which enables them to anticipate and adapt to periodic changes in the environment. In mammals, this time keeping mechanism, also known as the circadian system, is made up of a hierarchy of oscillators. A central clock in the suprachiasmatic nucleus of the hypothalamus coordinates peripheral clocks in multiple tissues^1^. The intracellular gene regulatory network of both the central and peripheral circadian clocks involves a relatively small set of master transcription factors (TFs) interconnected through multiple negative and positive feedback loops^2^. The core activators of the circadian network, the Clock Locomotor Output Cycles Kaput (CLOCK) and brain and muscle ARNT Like 1 (BMAL1), transcription factors from the basic helix–loop–helix (bHLH) family form a heterodimer complex CLOCK–BMAL1. In the absence of CLOCK, the Neuronal PAS domain protein 2 (NPAS2) which is also a member of the basic helix–loop–helix (bHLH)-PAS transcription factor family can compensate for the loss of CLOCK to form a heterodimer protein with Bmal1 to regulate the circadian clock^3^. In the classical model of clock gene regulation, the CLOCK–BMAL1 or NPAS2–BMAL1 dimer binds to a hexanucleotide sequence known as the E-box motif (canonical sequence CANNTG, where N is any nucleotide) within the promoter or enhancer regions of clock-controlled genes to regulate their transcription^3,4^. BMAL1 has also been shown to bind to E-box-like sequences, such as CACGTT in the promoter of the murine Per2 gene^5^. However, the experimental support for genome wide binding of BMAL1 to such sequences is lacking. Therefore, in this publication we have focused solely on the classical E-box with the sequence of CANNTG. Alterations in the expression or binding activity of the core clock TFs disrupt natural circadian oscillations, and can lead to numerous pathologies including insomnia, cancer, cardiovascular disease, and metabolic disorder^6,7^. Here we attempt to improve our understanding of gene regulation by the CLOCK–BMAL1 or NPAS2–BMAL1 complex and its perturbation using interpretable predictive models of DNA binding by the master regulatory factor BMAL1.

Genome-wide identification of transcription factor binding sites (TFBS) is a challenging problem. Typically, only a small fraction of classically defined sequence motifs for a particular TF are bound^8^. For example, the canonical E-box binding motif occurs more than 7 million times across the mouse genome, but less than 0.7% of these motifs are bound by CLOCK–BMAL1 or NPAS2–BMAL1 in mouse peripheral tissues^9^. Binding of a particular TF to its cognate DNA motif depends on several molecular features including the DNA sequence of the core motif, sequences flanking the core motif, chromatin accessibility, local shape of the DNA, presence of co-factors, histone modifications, DNA methylation, and other biophysical parameters^10–13^. These features and their relative contribution to binding can vary greatly across cell and tissue types^14,15^. Chromatin immunoprecipitation followed by sequencing (ChIP-seq) is the current gold standard for assaying genome-wide TF binding locations^16^. However, assaying the binding of a given TF under various conditions and in different tissues is prohibitively expensive. As such, several predictive computational models of genome-wide TF-DNA binding have been developed. From these models, DNA sequence and chromatin accessibility emerge as the most important determinants of TF binding^17–19^. Chromatin accessibility assays such as deoxyribonuclease hyper-sensitive sites sequencing (DNase-seq), and assay for transposase-accessible chromatin sequencing (ATAC-seq) have been used to improve TFBS prediction^20^. Recently, improved model predictions for TF binding have been obtained by leveraging advancements in machine learning and specifically deep learning techniques^21–23^. However, these models are often difficult to interpret and thus offer limited insights into the mechanisms governing the tissue specificity of TF-DNA binding.

In this study, we present interpretable machine learning-based models capable of predicting which canonical E-box motifs occurring in accessible chromatin regions of the mouse liver, heart, and kidney are likely to be bound by BMAL1. Our predictive models are based on the XGBoost machine learning algorithm^24^, with logistic regression used as a baseline algorithm to evaluate model performance. Published data from a BMAL1 ChIP-seq study^9^ was used to train and evaluate the models. When considering which features to include in our predictive models, we noted that DNA shape^25^ and histone modifications^26^ have been shown to be efficient predictors of TF binding in addition to DNA sequence. Specifically, it has been proposed that TFs prefer specific 3D DNA conformations and not just specific sequences^27^. For example, incorporation of DNA shape features led to improved model performance when predicting in vivo binding of TFs from the basic helix–loop–helix (bHLH) family^25^. Particularly, five distinct shape features—electrostatic potential (EP), minor groove width (MGW), propeller twist (ProT), roll, and helix twist (HelT) have been shown to be useful for TF-DNA binding prediction^28^.

Interpreting the structure of our models, we identified genomic and epigenomic features most predictive of BMAL1–DNA binding. Most of the flanking DNA sequence features showed low importance in predicting the binding of BMAL1, except the second flanking nucleotide upstream of the E-box motif in the liver. On the other hand, the histone modifications H3K27ac, H3K4me1, H3K4me3, H3K36me3, together with DNA shape features EP, Roll, and MGW were significant predictors of BMAL1–DNA binding in all tissues, resulting in high performing models. However, our cross-tissue predictive model showed that that even though there is high specificity for BMAL1 to bind certain DNA conformations and chromatin contexts, these specificities vary across tissues.

## Methods

### ChIP-seq dataset preprocessing

Uniformly processed BMAL1 ChIP-seq peaks from the C57BL/6J mouse liver, kidney and heart were obtained from Gene Expression Omnibus under the accession code GSE110604^9^. BMAL1 ChIP-seq experiments were performed at Zeitgeber time 6 (ZT6). The locations of accessible chromatin regions in DNase I-hypersensitive (DHS) sites for all three tissues (DNase-seq) were obtained from the Encyclopedia of DNA Elements, ENCODE (Supplementary Materials). The DNase-seq experiments were perfomed on unsynchronized tissues. The Genome Reference Consortium Mouse Build 38 (GRCm 38) was used as the reference genome. DHS sequences were processed in Python with BEDTools^29^ to extract all E-Box sequences (CANNTG) in accessible chromatin. E-box motifs in accessible chromatin regions but not overlapping their respective tissue ChIP-seq bed files were used as instances of unbound motifs (the negative dataset for the model). All accessible chromatin singleton E-boxes (instances of only one E-box motif under a BMAL1 peak) and E-boxes that were closest to the summit of the BMAL1 peaks for peaks with multiple E-boxes were labeled as bound (the positive dataset). All other E-boxes under BMAL1 peaks were considered ambiguous and ignored in further analysis. 1175 E-boxes, 1082 E-boxes, and 663 E-boxes from the bound Bmal1 liver, kidney and heart respectively were found to be ambiguous due to multiple E-boxes. We extended each E-box motif sequence to include 4-basepair (bp) flanking sequences upstream and downstream of the E-box. Since the E-box motif sequence is a palindrome, the reverse complement was ignored.Each E-box, thus represented by a 14-nucleotide sequence (6-bp core plus 4-bp sequence on either end), was one-hot encoded. The binary (bound and unbound) E-box data produced highly imbalanced datasets, as there were far more unbound than bound E-boxes in the mouse accessible chromatin. The bound E-box motif in the liver, kidney and heart numbered 3725, 3237 and 1313 respectively. The unbound E-box motif in the liver, kidney and heart numbered 189,581, 262,053 and 291,840 respectively. Specifically, the negative samples outnumbered the positives by factors of 51 in the liver, 223 in the heart, and 82 in the kidney. Like the previous reported occupancies of the number E-Box binding motif and the percentage of the motifs that are bound, the negative and positive samples reported in liver, kidney and heart are consistent with that.

### DNA shape preprocessing

Because of the degrees of freedom of the DNA sugar phosphate backbone, neighboring base pairs and bases within a pair can vary their position relative to each other causing a change in the shape of the DNA either through rotation or translation. We used the R/Bioconductor package DNAshapeR^30^ to estimate DNA shape features. The DNAshapeR algorithm predicts DNA shape features given a DNA sequence and encodes them in feature vectors. The feature vectors for each shape category were normalized to values between 0 and 1 by Min–Max normalization and placed in groups of 10 values for MGW, ProT and EP and groups of 11 values for HelT and Roll to be used as inputs for the predictive models. The number of bins for each shape feature is based on the length of the sliding window used to generate the features—5 bp for MGW, ProT, EP, and 6 bp for HelT and Roll.

### Histone modification preprocessing

We downloaded ChIP-seq data for five histone modifications, H3K27ac, H3K4me1, H3K4me3, H3K27me3 and H3K36me3, for mouse liver, kidney and heart tissues from ENCODE (Supplementary Materials). Histone modification ChIP-seq was performed on unsynchronized tissues. These histone modifications were chosen based on data availability for all tissues and their established roles in transcription factor binding. The corresponding bed files were used to generate signal profiles and heatmaps using deepTools^31^. From the profiles and heatmaps generated, we found the histone modification ChIP-seq signals extended meaningfully to at most 1.5-kb region (± 750 bp) centered on the E-box core motif. Using the 1.5-kb region centered at the E-box core motif, we extracted the histone modification features for the binary dataset for each tissue using bwtool^32^. The features were then divided into ten bins with the same number of nucleotides in each bin.

### Machine learning models

#### XGBoost

Extreme Gradient Boosting (XGBoost) is an ensemble learning method based on boosting trees for classification and regression^24^. We used up to 20 features as inputs for each E-box motif—ten sequence features (one for each nucleotide), five DNA-Shape features and five histone modification features. The first two and last two nucleotide of the E-box motifs were set because they were the same in all motifs. Using the Scikit-learn library, we performed hyperparameter tuning of the following parameters to reduce the degree of overfitting—the number of iterations in training (*n_estimators*), the sum of sample weight of the smallest leaf nodes to prevent overfitting (*min_child_weight*), the maximum depth of the tree in building a model while training (*max_depth*), the sampling rate of the training set in each iteration (*subsample*), the learning rate (*learning_rate*), and the feature sampling rate when constructing each tree (*colsample_bytree*). The hyperparameter tunning of the XGBoost model through a grid search of the hyperparameter space with the following values: *n_estimators* = {30, 40, 50, 60, 70, 80, 90, 100},* min_child_weight* = {1, 2, 3, 4, 5, 6},* subsample* = {0.5, 0.6, 0.7, 0.8, 0.9, 1 }, *max_depth* = {1, 2, 3, 4, 5},* learning_rate* = {0.1, 0.2, 0.3, 0.4, 0.5} and* colsample_bytree* = {0.6, 0.7, 0.8, 0.9, 1 } leading to a possible combination of 36,000 hyper-parameters.*.* In addition to tunning of the hyper parameters of the model, we also evaluated the model performance using fivefold cross validation on predicting the binding status of E-box motifs in accessible chromatin.

### Logistic regression

Logistic regression is a parametric classification model that estimates the probability that the output variable belongs to the appropriate class^33^. Logistic regression is used as the baseline for most machine learning-based classification models. In this study, we tuned the following logistic regression model hyperparameters to reduce overfitting in our testing dataset—the regularization solver for the training dataset (*solver*), and the maximum number of attempts the solver algorithm is to run before it converges (*max_iter*).

## Results

### BMAL1 binds most frequently to the CACGTG E-box motif in all tissues

**Figure 1:**
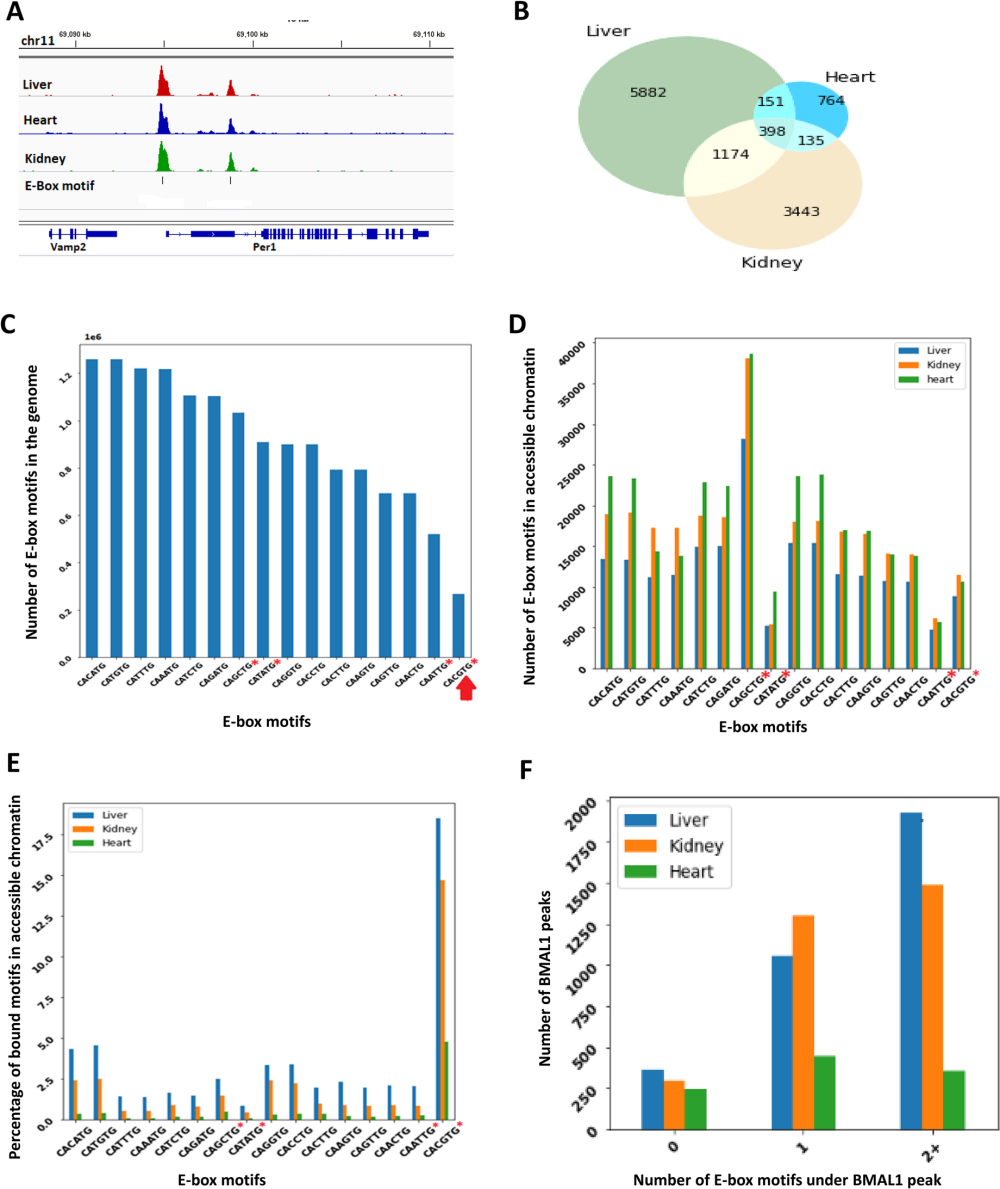
Binding of BMAL1 to E-Box motifs across multiple mouse tissues. **(A)** BMAL1 ChIP-seq peaks in the liver (red), kidney (blue), and heart (green), and E-box binding motifs (black vertical bars) under the peaks at the Per1 locus. **(B)** Venn diagram representing the overlap of bound E-boxes motifs in open chromatin across liver, kidney and heart. **(C)** E-box binding motif distribution across the entire mouse genome. The canonical E-Box motif CACGTG (marked with an arrow) is the least represented motif in the mouse genome. **(D)** Distribution of E-box binding motifs in open chromatin across the liver (blue), kidney (orange) and heart (green). **(E)** Percentage of BMAL1 bound E-box motifs in open chromatin across the liver (blue), kidney (orange) and heart (green). **(F)** Distribution of BMAL1 peaks with zero (0-E-Box), exactly one (singleton E-box) and multi (two or more E-box) E-box motifs in the liver (blue), kidney (orange) and heart (green). In (**C–E**) the sequences along the x-axis are ordered by their frequency in the mouse genome shown in (**C**), which also happens to group complementary sequences adjacent to each other. The four palindromic sequences, which are their own complements, are marked with asterisks.

BMAL1 is known to bind to E-box motifs, and these motifs are considered to have a consensus sequence of **CANNTG**^34^.Therefore, we scanned the mouse mm10 reference genome and identified instances of the canonical E-Box motif (**CANNTG**). Since we have investigated all possible nucleotide permutation of the central two nucleotides, the reverse complement of the canonical E-box sequence was considered but a particulate E-box and its reverse complement were considered separately. For each DNase-seq dataset obtained from ENCODE for C57BL/6J mouse tissues (liver, heart, and kidney), we found the subset of E-boxes overlapping DNase-seq hypersensitive sites (DHS), i.e., E-boxes in accessible chromatin. Tissue specific lists of E-boxes in accessible chromatin were then compared with their tissue matched BMAL1 ChIP-seq peaks^9^ to extract all BMAL1-bound and unbound E-Boxes in accessible chromatin. Additionally, we found instances where BMAL1-bound E-boxes were not located in accessible chromatin (0.8% of all peaks). We excluded these E-boxes from model training and evaluation, to avoid confounding between the two classes of bound E-boxes. First, we compared occurrences of BMAL1-bound E-boxes in accessible chromatin across liver, heart, kidney and observed that they were highly tissue-specific, with only 398 E-boxes bound in common in all three tissues (Fig. 1A,B). E-boxes bound in all three tissues were often found in promoters of core circadian clock genes (results not shown). Next, we counted all instances of the canonical E-box motif (CANNTG) in the mouse genome, where *N* represents any nucleotide type. The canonical E-box includes 16 distinct E-box types, one for each permutation of the **NN** dinucleotide in the center of the motif. We computed the fraction of each individual E-box type compared to the total number of E-boxes (Fig. 1C). The E-box types **CACATG** and **CATGTG** represented the highest fraction of E-boxes in the mouse genome, jointly comprising 17.3% of all instances. These two motifs are the reverse complements of each other, and like all other non-palindromic E-boxes that are reverse complements of each other, the two show roughly equal frequencies. Interestingly, the palindromic BMAL1-preferred E-Box motif, **CACGTG**, occurs the fewest number of times (1.83% of all instances) in the mouse genome (Fig. 1C).

We then applied the same procedure to E-boxes in accessible chromatin of mouse liver, kidney, and heart. The palindromic motif **CAGCTG** was the most common E-box type across accessible chromatin regions in all three tissues, while the BMAL1-preferred E-Box **CACGTG** was among the three least common motifs which were all palindromes (Fig. 1D). We then used the overlap between the BMAL1 ChIP-seq and DNase-seq peaks to compute the percentage of BMAL1-bound E-boxes in the mouse liver, kidney, and heart, relative to the total number of E-boxes of the same type in accessible chromatin of their respective tissue. The BMAL1-preferred E-box **CACGTG** was the most frequently bound E-box type across all three tissues. In addition, about 18% of **CACGTG** E-boxes accessible in the liver were also bound in the liver, and for the kidney and heart these fractions were 15%, and 4%, respectively. Furthermore, less than 20% of all individual E-boxes found in accessible chromatin in any particular tissue were also bound in that same tissue (Fig. 1E). The kidney and heart had a higher number of E-boxes in accessible chromatin when compared to the liver. However, the liver had a higher number of BMAL1-bound E-boxes.

We observed instances where there were none (zero), exactly one (singleton) and two or more (multi) E-box motif(s) under a single BMAL1 ChIP-seq peak in all tissues (Fig. 1F). We then extracted all singleton E-boxes and E-boxes closest to the summit of the BMAL1 peak within multi-E-box peaks and labeled these as bound (positive dataset). The E-Boxes in accessible chromatin that were not bound by BMAL1 were labelled as unbound (negative dataset). All other E-boxes were left out from further analysis. The ratios of the positive to negative datasets were 1:51, 1:82, and 1:223 in liver, kidney, and heart, respectively.

Together, these results indicate that BMAL1 likely interacts, in a tissue-specific manner, with multiple different E-box types across the liver, kidney, and heart, with **CACGTG** being the most highly associated with BMAL1 binding. 

### Predicting genome wide BMAL1 binding within tissues

**Figure 2:**
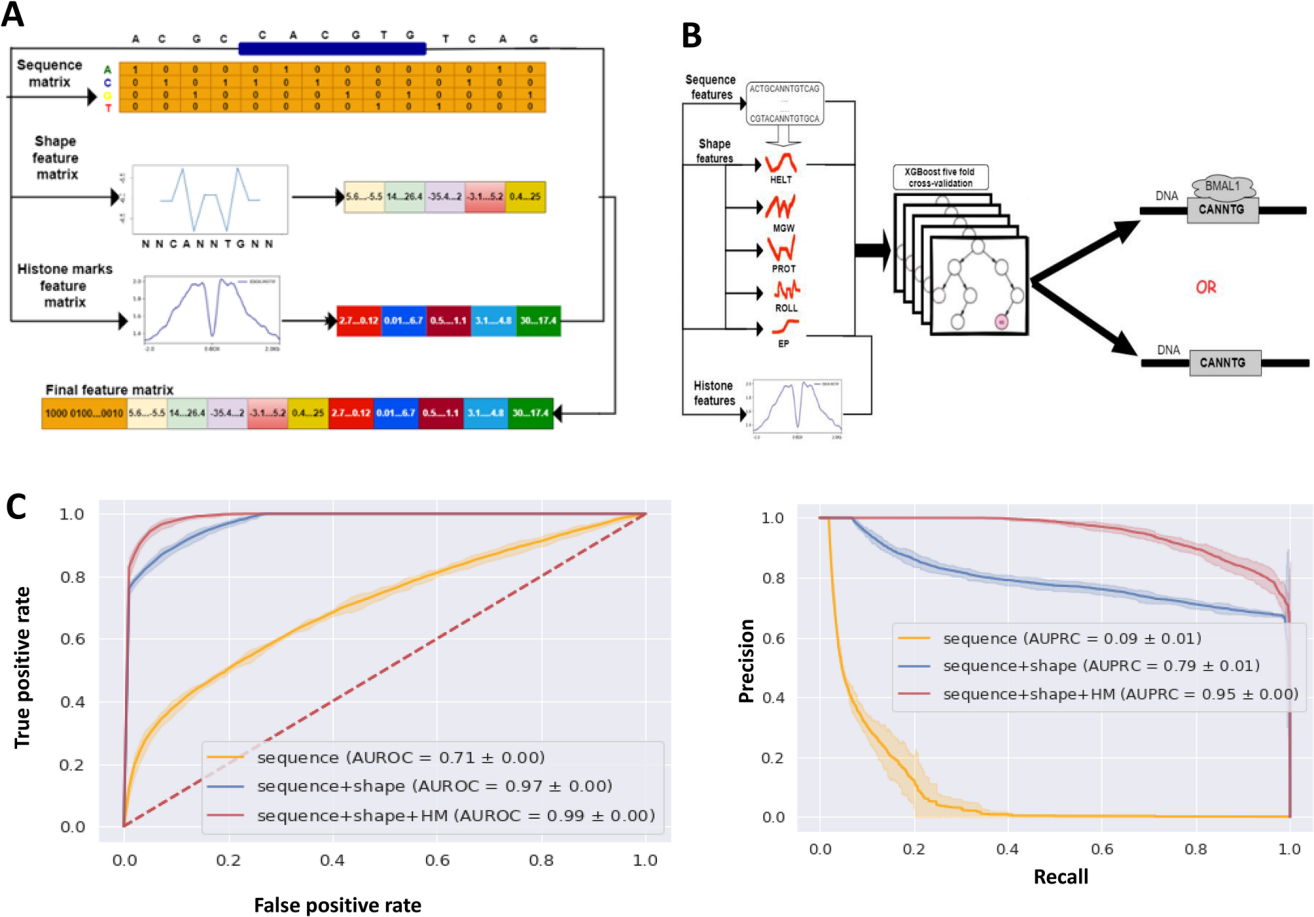
Machine learning model predicting BMAL1 binding to E-box motifs across three mouse tissues. **(A)** Design of the machine learning algorithm input features. The local chromatin features (E-box DNA sequence features) and flanking sequences were one-hot encoded. The DNA shape genomic feature matrix from the k-mer + k-shape (k = 1) sequence feature model and epigenomic (histone modification) features averaged and binned were used as the final feature matrix for the model (generated using diagrams.net v21.1.2: https://app.diagrams.net/?src=about). **(B)** Schematic of the machine learning- predictive model. Based on fivefold cross-validation, the XGBoost classifier predicted the binding status of E-box motifs in open chromatin, training on all accessible bound E-boxes and unbound E-boxes (generated using diagrams.net v21.1.2: https://app.diagrams.net/?src=about). **(C)** Performance of models predicting the binding status of E-boxes in open chromatin of the liver. The performance of each model is represented as a mean line with a shaded 95% confidence interval from fivefold cross-validation. The legend shows the list of features used an area under the curve. Both receiver operating characteristic (ROC) and precision-recall (PRC) curves showed progressive improvement in model prediction with addition of genomic and epigenomic features.

Nucleotides flanking the E-box have been shown to affect the binding specificity of an E-box binding TFs^18^. Therefore, we extended and one-hot encoded the genomic sequence for all BMAL1-bound (positive) and unbound (negative) E-boxes by 4 bps up- and down- stream of the E-box (Fig. 2A). Additionally, we computed the following DNA shape features for the extended, 14 bp sequence—electrostatic potential (EP), minor groove width (MGW), propeller twist (ProT), roll, and helix twist (HelT), using the k-mer + k-shape (k = 1) sequence feature model^13^ (Fig. 2A). Even though the shape features are derived from DNA sequence, they can potentially capture high order interdependencies between neighboring nucleotide and thus add extra information to the model input. DNA shape features can also explain the importance of flanking sequence in TF-DNA binding specificity^18^. Visualization of the DNA shape features EP, ProT, and Roll showed differences in DNA shape between the bound and unbound motifs across the liver, kidney, and heart, while the MGW feature showed a difference between the bound and the unbound motifs for the kidney only (Supplementary Figs. 1–3). The shape feature vector for each category was then normalized to values between 0 and 1 using Min–Max normalization and binned in groups of ten values for the DNA shape features EP, MGW and ProT, and groups of 11 values for HelT and Roll. These normalized DNA shape feature vectors were used as input features for the predictive models as shown in Fig. 2A.

Epigenetic modifications are also known to influence transcription factor binding. Specifically, histone modifications are involved in regulation of transcription factor occupancy and subsequent regulation of gene expression^25,35^. Histone modification ChIP-seq binding signal values, across the genomic regions spanning ± 750 bps around the E-box were used to compute feature vectors for five histone modifications: H3K27ac, H3K4me1, H3K4me3, H3K27me3 and H3K36me3. The ± 750-bp region was chosen to consider local profiles of histone modifications around the size of a typical promoter or enhancer. The histone feature vector was binned into ten bins with the signal strength averaged across 150 bps of each bin (Fig. 2A)^36^.

We implemented three different models using subsets of the final encoded feature set: (i) DNA sequence-only; (ii) DNA sequence and DNA shape (sequence + shape); and (iii) DNA sequence, DNA shape, and histone modification (sequence + shape + HM) model^36^. We used two machine learning algorithms to predict the binding status of E-boxes in accessible chromatin. XGBoost^24^ was our principal predictive algorithm, and we compared its performance with that of a baseline logistic regression model. Using grid search and stratified fivefold cross validation, we tuned model hyperparameters and derived the optimal hyperparameters for each model based on the liver, heart, and kidney datasets. The model with the optimal hyperparameters was trained through five-fold stratified cross validation and used to predict the binding of BMAL1 to the E-boxes in the liver, heart and kidney; and average performance across the 5 folds was reported (Fig. 2B,C). Model performance was evaluated using the performance metrics—area under the receiver operating characteristic (AUROC) and area under the precision-recall curve AUPRC, which showed that XGBoost outperformed logistic regression (Table 1, Supplementary Figs. 4, 5).

**Table 1:** Model performance scores: the performance of models predicting BMAL1–DNA binding status in open chromatin of the liver, kidney, and heart using XGBoost and logistic regression.

	XGBoost		Logistic regression	
	AUROC	AUPRC	AUROC	AUPRC
Liver				
DNA sequence only model	0.71 ± 0.00	0.09 ± 0.01	0.71 ± 0.00	0.08 ± 0.01
DNA sequence plus DNA shape model	0.97 ± 0.00	0.79 ± 0.01	0.97 ± 0.00	0.79 ± 0.01
DNA sequence plus histone modification model	0.85 ± 0.02	0.13 ± 0.01	0.80 ± 0.00	0.12 ± 0.03
DNA shape plus histone modification model	0.90 ± 0.01	0.22 ± 0.01	0.81 ± 0.01	0.16 ± 0.01
DNA sequence and shape plus histone modifications model	**0.99 ± 0.00**	**0.95 ± 0.00**	0.97 ± 0.00	0.91 ± 0.00
Kidney				
DNA sequence only model	0.78 ± 0.01	0.10 ± 0.01	0.77 ± 0.00	0.10 ± 0.01
DNA sequence plus DNA shape model	0.94 ± 0.00	0.50 ± 0.01	0.79 ± 0.01	0.10 ± 0.01
DNA sequence plus histone modification model	0.89 ± 0.00	0.19 ± 0.01	0.87 ± 0.00	0.13 ± 0.01
DNA shape plus histone modification model	0.95 ± 0.01	0.31 ± 0.01	0.88 ± 0.01	0.15 ± 0.01
DNA sequence and shape plus histone modifications model	**0.96 ± 0.00**	**0.65 ± 0.01**	0.88 ± 0.01	0.15 ± 0.01
Heart				
DNA sequence only model	0.80 ± 0.01	0.06 ± 0.01	0.78 ± 0.01	0.05 ± 0.01
DNA sequence plus DNA shape model	0.99 ± 0.00	0.71 ± 0.03	0.97 ± 0.01	0.49 ± 0.02
DNA sequence plus histone modification model	0.96 ± 0.00	0.26 ± 0.01	0.92 ± 0.01	0.8 ± 0.01
DNA shape plus histone modification model	0.97 ± 0.00	0.35 ± 0.00	0.95 ± 0.00	0.22 ± 0.00
DNA sequence and shape plus histone modifications (Heart)	**0.99 ± 0.00**	**0.80 ± 0.04**	0.98 ± 0.01	0.47 ± 0.02

Performance of each model is represented as a mean value with a 95% confidence interval around the results from fivefold cross validation. The highest model performance for each tissue is bolded.

### DNA shape and histone modification features improve within tissue model performance

#### Performance of sequence-only models

**Figure 3:**
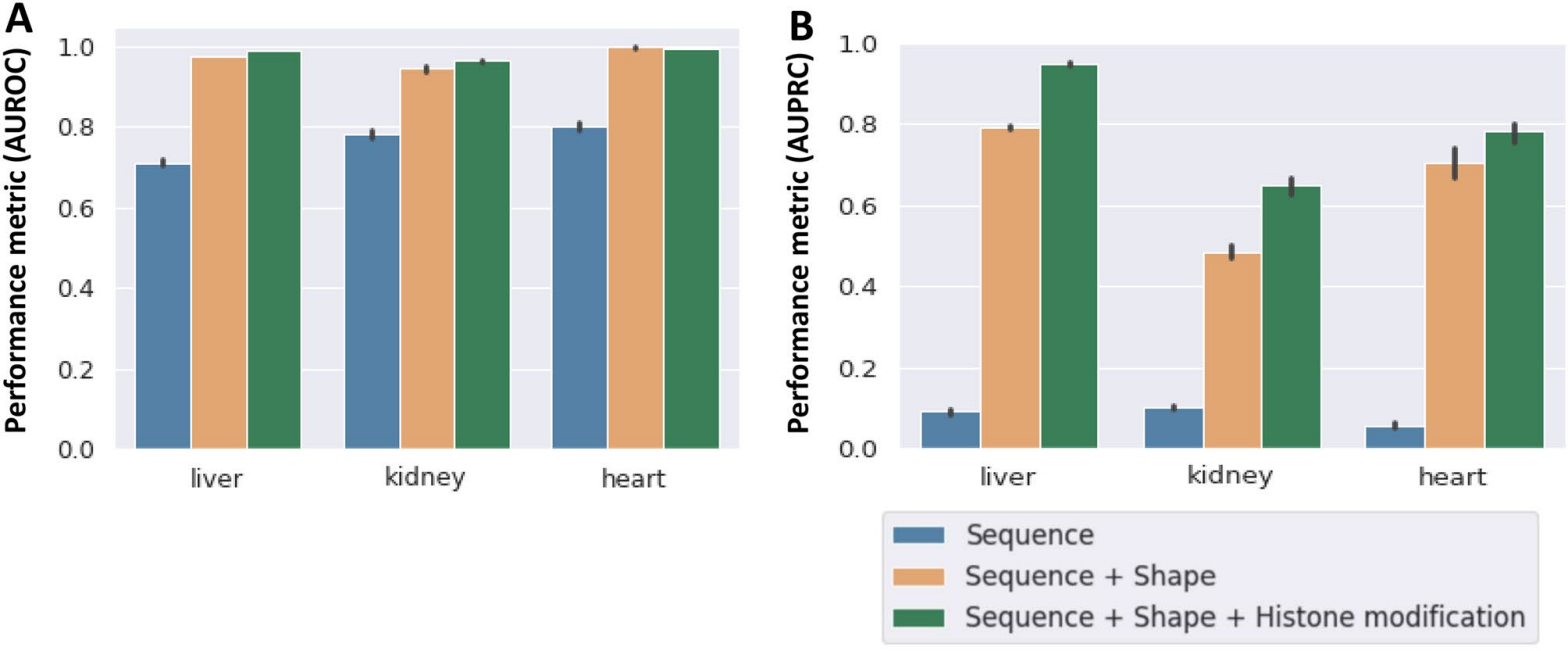
Adding DNA Shape and Histone modification (HM) features to DNA Sequence significantly improves prediction of BMAL1 binding across all tissues. **(A)** The area under the receiver operating characteristics (AUROC) for liver, kidney, and heart for the sequence-only model (blue), sequence plus DNA shape model (brown) and sequence plus DNA shape plus HM model (green). The mean AUROC increases sharply with the addition of DNA shape features to the model, with a much smaller increase associated with the addition of HMs. **(B)** The area under the precision recall curve (AUPRC) in liver, kidney and heart for the sequence-only model (blue), sequence plus DNA shape model (brown) and sequence plus DNA shape plus HM model (green). As with AUROC, the mean AUPRC increased by a large margin with the addition of DNA shape features to the model, with a smaller increase associated with the addition of HMs.

In order to derive mechanistic insights, we developed two interpretable machine learning models based on logistic regression (LR) and XGBoost algorithms. LR was used as our baseline model. We trained and validated our XGBoost classifier on the liver, heart, and kidney with ten sequence features comprising two central nucleotides of the E-Box and an additional four flanking nucleotides up and down- stream of the E-box (NNNNCANNTGNNNN where the conserved CA and TG subsequences are not included). We calculated the average AUROC and AUPRC scores for each tissue using stratified fivefold cross-validation. The AUPRC can be considered a more appropriate metric in our case, given the unbalanced distribution in the two classes—bound vs unbound E-boxes. The mean AUROC scores were 0.71, 0.78, and 0.80 for the liver, kidney, and heart respectively (Fig. 3A), with corresponding mean AUPRC scores of 0.09, 0.10, and 0.06, respectively (Fig. 3B). The relatively high AUROC and AUPRC scores across all tissues suggest differences in the two central nucleotides and flanking sequence between BMAL1 bound and unbound E-boxes. However, there does not appear to be sufficient information in DNA sequence alone for a robust prediction.

#### Performance of sequence + shape models

The three-dimensional structure of DNA gives rise to specific local conformations. Features to quantify DNA shape have been derived computationally using Monte Carlo simulations from local DNA sequence^28,37^. Five DNA shape features—electrostatic potential (EP), minor groove width (MGW), propeller twist (ProT), roll, and helix twist (HelT) were found to contribute to the binding affinity of transcription factors from the basic helix loop helix (bHLH) family^13^. We combined the DNA shape feature matrix with the sequence features as input for the model, to evaluate the contribution of DNA shape to BMAL1 binding. The mean AUROC scores were 0.97, 0.98, and 0.98 for the liver, kidney, and heart, respectively (Fig. 3A) which are all somewhat higher than the sequence-only model. Compared to the sequence-only model, the mean AUPRC metric increased sharply from 0.09 to 0.79 for the liver, 0.10 to 0.51 for the kidney, and 0.06 to 0.71 for the heart (Fig. 3B), suggesting significant differences in local DNA shape features between the bound and unbound E-boxes. Inspection of feature importance revealed that the EP, Roll and ProT DNA shape features contributed 33% to the prediction of BMAL1 binding to the E-boxes in the liver. For the kidney, the EP, ProT and MGW DNA shape features contributed 68% to prediction of BMAL1 binding, while in the heart, EP, Roll and MGW contributed 70% to the prediction. Overall, the EP, Roll, MGW and ProT DNA shape features had the biggest influence on prediction of bound E-Boxes across all three tissues (Supplementary Fig. 6). We also trained and evaluated DNA shape only models, however its performance was lower than even DNA sequence only models, suggesting that local shape or configuration of the DNA near the E-box by itself is not sufficient to predict BMAL1 binding (results not shown).

#### Performance of sequence + shape + histone modification (HM) models

HMs in gene promoter and enhancer regions are known to be correlated with transcription factor (TF) binding^38^. However, the mechanisms of interaction between TF binding and HMs are not fully understood. Recent studies have shown that the extent to which HMs improve the performance of models predicting TF binding is TF-specific, with models of bHLH transcription factor binding showing significantly improved accuracy when HMs are included^39,40^ Based on these findings, several models have been developed to improve TF binding prediction using results from epigenetic assays^41,42^. We examined the importance of HMs in prediction of BMAL1 binding by adding five histone features (H3K27ac, H3K4me1, H3K4me3, H3K27me3 and H3K36me3) to the sequence and DNA shape feature matrix. These HM features were chosen based on data availability and their roles in transcription factor binding described in literature^40^. Using models incorporating HM features, we obtained mean AUROC scores of 0.99, 0.988 and 0.99 for the liver, kidney, and heart, respectively (Fig. 3A). The mean AUPRC performance increased significantly to 0.95, 0.65 and 0.79 for the liver, kidney, and heart respectively (Fig. 3B).

### Feature importance reveals tissue-specific BMAL1 binding grammar

**Figure 4:**
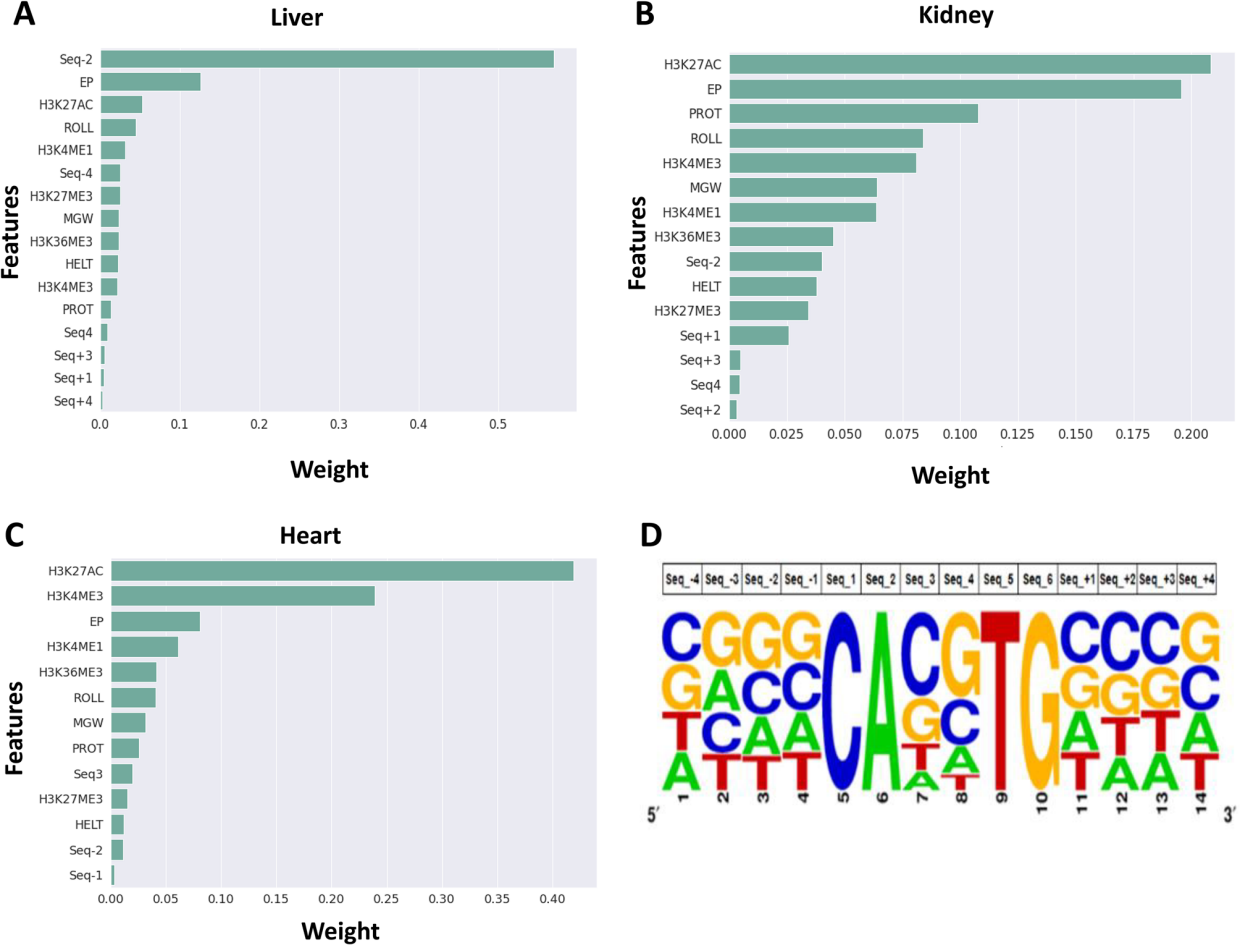
Feature importance of all genomic features (sequence and DNA shape) and epigenomic (histone modification) features from the XGBoost classifier model across all tissues. Feature importance in the XGBoost classifier model in **(A)** liver, **(B)** kidney, and **(C)** heart. The feature importance for each DNA shape and histone modification feature is calculated as the sum of all the feature importance of all bins for that particular histone modification feature. The feature importance of each nucleotide type at a particular position relative to the E-Box motif is normalized to the nucleotide type and the sum of all feature importance at that nucleotide position. **(D)** Standard plot sequence logo for BMAL1 bound E-box motifs in the liver^44^.

**Figure 5:**
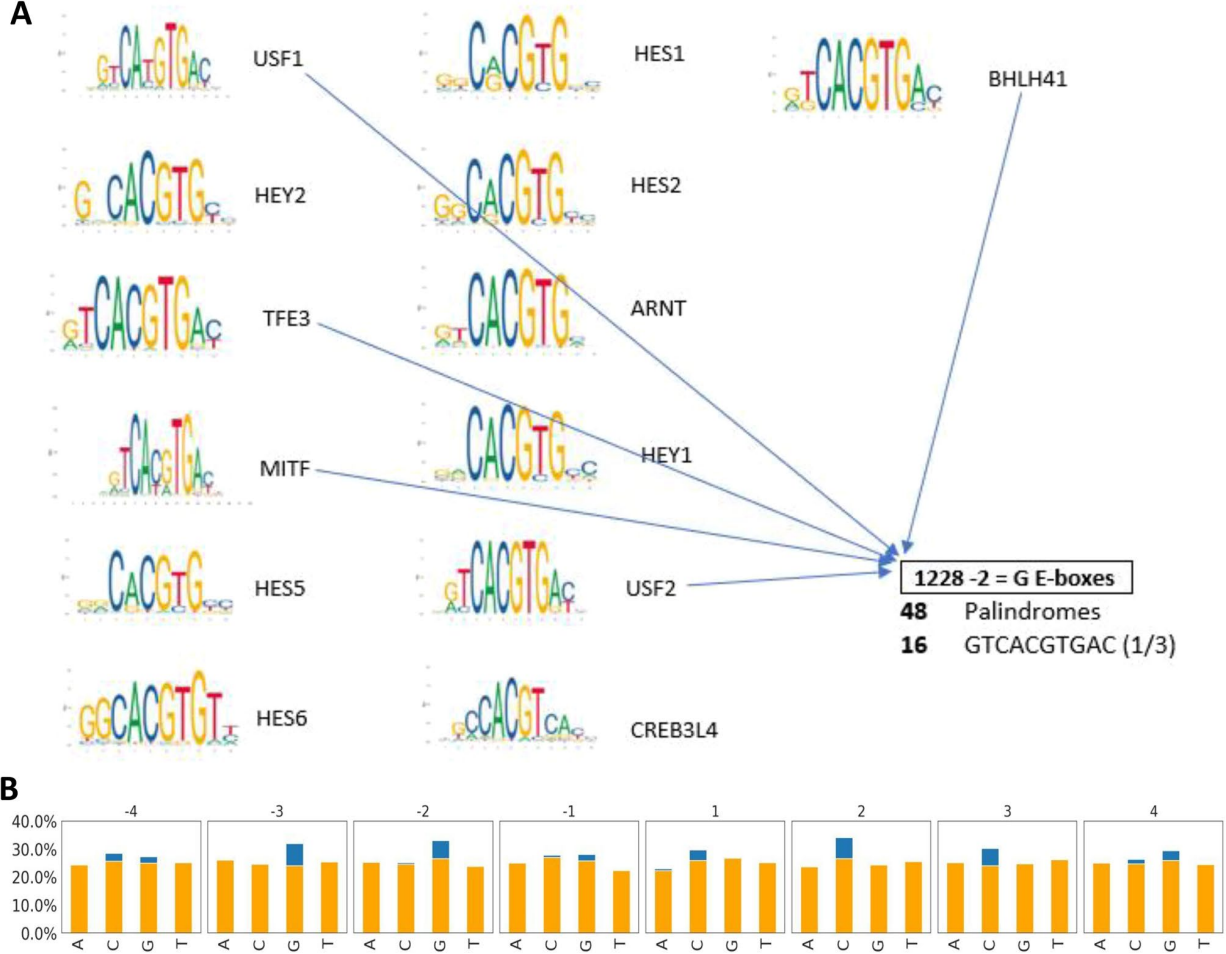
Analysis of Liver bound E-box motifs to investigate the importance of the nucleotide G in the third position of the 5’ flanking sequence. **(A)** Analysis of the bound E-box motif with their upstream and downstream flanking sequence revealed that the nucleotide **G** at the third position of the 5’ flanking sequence is enriched in bound E-box motifs in the liver. 1228 out of 3374 of the motifs have nucleotide **G** at the third position of the 5’ flanking sequence. 48 out of the 1228 are palindromes and 16 out of the 48 are the sequence **GTCACGTGAC. (B)** Percentage of enriched flanking sequence nucleotide in the liver E-box motifs (orange bar corresponds to unbound E-box motifs and blue bar corresponds to bound E-box motifs).

Given the improved performance of the sequence + shape + HM models, we used the ELI5 permutation importance method^43^ to identify features most predictive of BMAL1–DNA binding. The importance for each DNA shape and histone modification feature was calculated as the sum of the importance of all bins for that particular feature. The feature importance of each nucleotide type at a particular position relative to the E-Box motif was normalized to the sum of all feature importance at that nucleotide position. The immediate flanking sequences upstream and downstream of the core E-box binding motif were important predictors of BMAL1 binding in the liver, heart and kidney as compared to distal flanking sequences (Fig. 4). Analysis of the binding specificities of the bHLH transcription factors CBf1 and Tye7 in yeast has previously shown that 2-bp flanking sequences contribute to binding of these transcription factors to the E-box^18^. In our quantitative analysis of the E-box sequence, we did not find the two central base pairs of the CANNTG E-box motif to directly contribute to the model performance across the three mouse tissues, even though BMAL1 has a strong preference for the **CG** central dinucleotide across all three mouse tissues. Analysis of the feature weights showed the nucleotide **G** at the second proximal upstream flanking sequence (Seq-2) to be a strong predictor of BMAL1–DNA binding in the liver (Fig. 4A). This nucleotide accounted for more than 50% of feature weights used in predicting BMAL1–DNA binding in the liver. Other contributing features included EP (10%) and H3K27ac (6%). Most of the DNA shape and histone modification features had weights greater than 5% indicating their importance in predicting BMAL1 DNA binding in the liver, while most of the DNA sequence features except Seq-2 had a feature weight of less than 5%. In the kidney, H3K27ac had the highest feature importance, contributing 21% to the overall feature importance (Fig. 4B). EP followed with a feature importance of 19%. Three histone modifications (H3K27ac, H3K4me3 and H3K4me1) and four DNA shape features (EP, ProT, MGW and Roll) all had feature weights > 5%. In the heart, H3K27ac and H3K4me3 had the highest feature importance (both > 20%) followed by EP (8%). Most of the DNA sequence features had weights < 5% in both heart and kidney. The histone modifications H3K27ac, H3K4me1, H3K4me3 and DNA shape features EP, and Roll showed high importance scores across all three tissues (Fig. 4A–C). The histone modifications with the largest contribution to BMAL1 binding were H3K27ac, H3K4me1 in all three tissues, and H3K4me3 and H3K36me3 in the kidney and heart. These results show that the combination of the TF binding motif and its flanking sequence, local shape of DNA, and histone modifications is sufficient to produce predictive models of BMAL1 binding to E-box motifs, especially in the mouse liver. The second upstream flanking nucleotide (Seq-2) had by far the highest feature importance score in the liver. The nucleotides **G** and **C** were overrepresented at the second proximal upstream flanking sequence of the liver bound E-box motifs. This was supported by the sequence logo of the bound E-box sequence with 4 bps upstream and downstream of the core E-box motif (Fig. 4D). Analysis of the bound E-box motifs along with their upstream and downstream flanking sequence revealed that the nucleotide **G** is enriched at the third position of the 5’ flanking region (1228 out of 3374 bound E-boxes in the liver) (Fig. 5A,B). This was not the case for bound E-box motifs in the kidney and heart.

### Cross-tissue models highlight differences in BMAL1–DNA binding in different tissues

**Figure 6:**
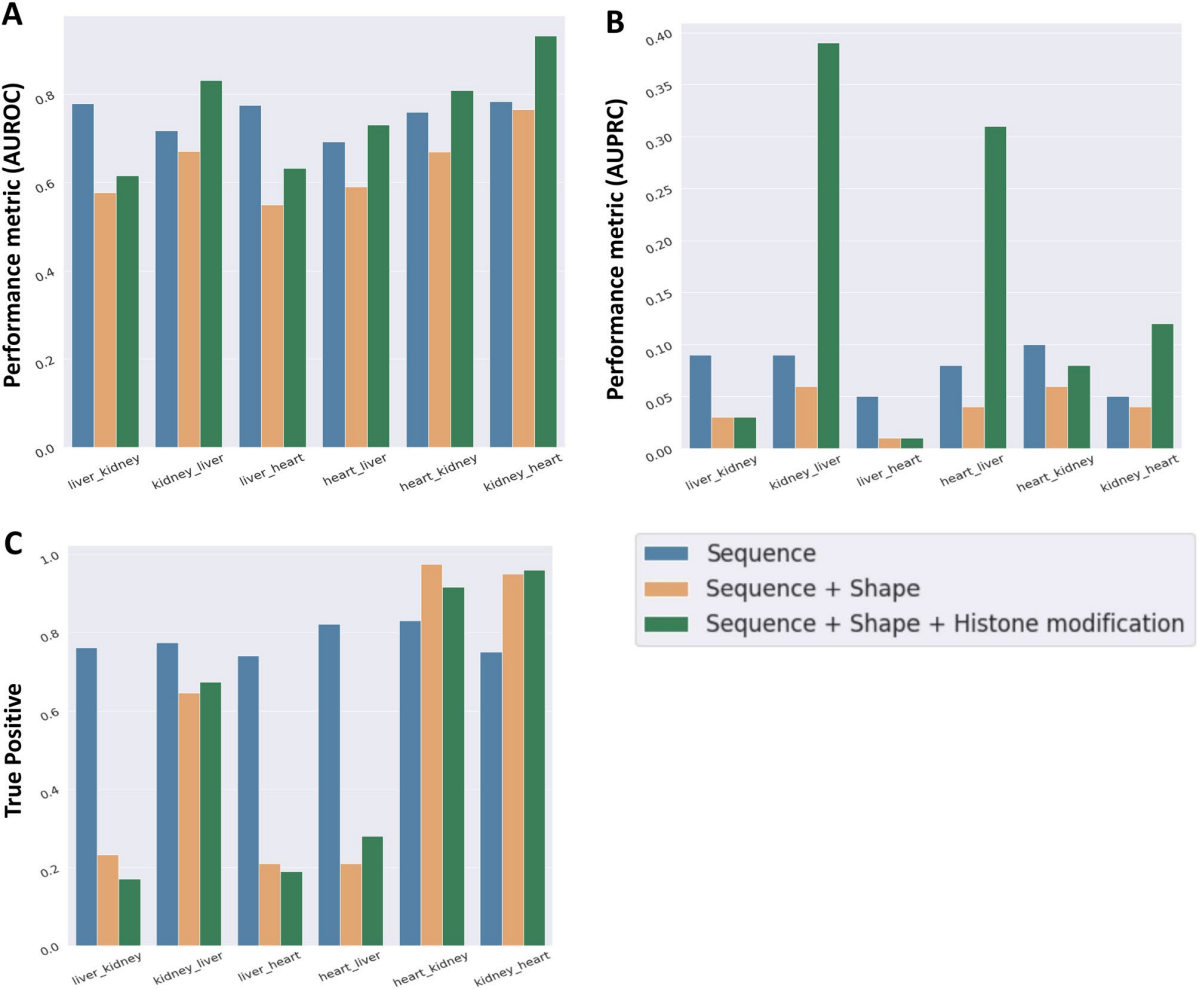
Performance metrics for cross-tissue prediction models. Scores for the liver, kidney and heart sequence-only model (blue bars), sequence plus DNA shape model (brown bars) and sequence plus DNA shape plus HM model (green bars)**: (A)** area under the receiver operating characteristics (AUROC); **(B)** area under the precision recall curve (AUPRC); **(C)** true positive rates (notation explanation: liver_kidney refers to the model trained on the liver dataset and used to predict binding on the kidney dataset).

To test the hypothesis that the DNA binding of BMAL1 is determined by similar factors across the three tissues, we developed cross-tissue models for binding prediction with features based on—(a) sequence only; (b) sequence plus DNA shape; and (c) sequence plus DNA shape plus histone modifications. We trained these models on all data available in the respective tissue, using the optimal hyper-parameters previously derived for the respective within-tissue model. Trained models were used to predict BMAL1 binding in a different tissue. Performance of the sequence-only models trained on tissue X and predicting tissue Y (X_Y model) was similar to the performance of the within-tissue sequence-only model in tissue X, for all tissues (Fig. 6A, blue bars). Surprisingly, the addition of the DNA shape and HM features resulted in decreased performance scores across all cross-tissue models relative to the sequence only models (Fig. 6A–C). The sequence plus shape model trained on the liver data was able to correctly classify 22% of the E-boxes bound in both kidney (liver_kidney) and heart (liver_heart) (Fig. 6C, brown bars). This model predicted most of the bound E-boxes in the kidney and heart as unbound, yielding a high false negative rate. The addition of histone modification features improved the AUROC and AUPRC for most cross-tissue models (Fig. 6A,B, green bars). However, the cross-tissue sequence plus DNA shape plus HM model trained on the liver data correctly classified only 18% of E-boxes bound in the kidney and 19% in the heart, also leading to a high false negative rate than the sequence plus shape model.

Interestingly, models trained on kidney and heart and evaluated on the liver (kidney_liver and heart_liver) displayed a dramatic increase in performance with the sequence plus DNA shape plus HM models compared to other types of models. The AUROC performance of the kidney_liver model increased from 0.68 for the sequence plus DNA shape model to 0.83 for the sequence plus DNA shape plus HM model, while the AUPRC score increased sharply from 0.055 to 0.38. In summary, while sequence-only models performed relatively poorly in cross-tissue binding prediction, adding genomic features like DNA shape and epigenetic features like histone modifications generally decreased the performance even further in most cross-tissue models. These results highlight the tissue specificity of BMAL1 DNA binding.

## Discussion

Identification of transcription factor (TF)-DNA binding determinants can improve our understanding of gene regulatory grammar^45^. However, precise DNA-binding sequences and the amount of flexibility in these sequences are currently unknown for many TFs, including BMAL1, a master regulator of the circadian clock. Features other than the simple DNA binding site sequence clearly contribute to usage of a DNA sequence as a TF binding site. While the architecture of the core clock gene regulatory network in the suprachiasmatic nucleus of the brain is believed to be similar to the architecture in peripheral tissues, clock-controlled gene expression is largely tissue-specific^9,46^. Here we used XGBoost, an ensemble decision tree-based machine learning algorithm, to predict the binding of BMAL1 to its putative binding motif (the E-box) in three mouse tissues—liver, heart and kidney. We developed three different types of models: (1) sequence-only, (2) sequence plus DNA shape, and (3) sequence plus DNA shape plus histone modifications (Fig. 2A,B).

The **CACGTG** E-box type showed up the fewest number of times, compared to other E-box types, in the whole mouse genome and in accessible chromatin regions of all three tissues (Fig. 1C-D). However, this E-box type was the most frequently BMAL1 bound (Fig. 1E). This is consistent with the observations that the BMAL1-preferred binding motif is **CACGTG**^47^. Interestingly, even though the dinucleotide **CG** was over-represented at the center of the BMAL1-bound E-boxes, these nucleotides did not enhance model performance. Additionally, the heart had more E-boxes in accessible chromatin than the liver and kidney; but had the lowest number of bound E-boxes in accessible chromatin (Fig. 1D,E). The role of circadian rhythms in the heart is not well understood, and only 6% of protein coding genes in the mouse heart are circadian-regulated as compared to 11–16% in the liver^48^. Most likely, this is a consequence of lower overall BMAL1 binding in the heart. However, the low level of BMAL1 binding to otherwise accessible E-boxes in the heart remains to be resolved. One possibility is that a heart-specific E-box binding factor interferes with BMAL1 binding to E-boxes. For example, it has been shown that elevated levels of Usf1, a ubiquitous TF, can interfere with the binding of a mutant CLOCKΔ19:BMAL1 to E-box sites^49^, and other such factors likely exist. Interestingly, neither kidney nor heart within-tissue models achieved the same level of performance as the liver within-tissue model (Fig. 3A,B). There is evidence that the heart circadian rhythm might be phase-shifted when compared to the liver, indicating that the maximal BMAL1 binding in the heart might occur at a different time than the time shown to result in maximal BMAL1 binding in the liver—Zeitgeber time 6 (ZT06)^9^. Since the same time was used in the heart BMAL1 ChIP-seq experiment, this could result in some of the heart BMAL1-bound E-boxes being labeled as unbound, which would affect model learning and be reflected in lower model performance, as observed. A limitation of our work is that we had only considered E-boxes in accessible chromatin and disregarded inaccessible E-boxes. Our observations confirmed that on average more than 75% of BMAL1 peaks lie in accessible chromatin, therefore BMAL1 is more likely to bind in accessible chromatin. However, it has been demonstrated that BMAL1-CLOCK can act as a pioneering factor and rhythmically control the accessibility of chromatin surrounding the BMAL1 bound sites^50^.

Recent studies have shown that DNA shape computed using core TF binding motifs and their flanking sequences improves TF binding prediction for many human TFs^13,25,51^. Additionally, DNA topology is highly correlated with the structure and stability of the nucleosome, suggesting that topological changes can influence the binding of TFs to DNA^52^. In our sequence plus shape models the EP, Roll, MGW and ProT DNA shape features had the highest influence on prediction of bound E-boxes (Supplementary Fig. 6). A recent study^13^ showed that for Max, a basic helix–loop–helix (bHLH) TF like BMAL1 and CLOCK, it was Roll and ProT that were the dominant determinants of TF-DNA binding affinity. These observations agree with our findings. Further, in agreement with previous studies, we found that DNA shape features by themselves do not enhance model accuracy.

Analysis of feature importance of our sequence plus shape plus histone modifications (HMs) models showed that the HMs H3K27ac and H3K4me3, and the DNA shape feature EP dominated binding prediction across the kidney and the heart and were also ranked highly in the liver. It has been shown that H3 acetylation and methylation modifications surrounding CLOCK–BMAL1 bound sites change in a rhythmic fashion^53^. Our results demonstrate that even with only a snapshot of these HMs, i.e., a single ChIP-seq experiment from mice that are not light/dark synchronized, we can discern which E-boxes are bound and which are not with high levels of sensitivity. We propose that this is likely due to the information that is encoded in the shape and flanking sequence of the E-box motif in addition to the average levels of histone acetylation and methylation. Furthermore, we propose that this information is tissue specific as evidenced by the performance of our cross-tissue models. Intriguingly, the DNA sequence features by themselves had little to no effect on binding prediction in the kidney and heart. However, the second nucleotide upstream of the E-box had a large contribution to predicting BMAL1–DNA binding in the liver. The nucleotide **G** in this position contributed to about 50% of the feature importance score in the liver. Analysis of the bound E-box motif with their upstream and downstream flanking sequence revealed that the nucleotide **G** at the third position of the 5’ flanking sequence is enriched in bound E-box motifs in the liver Since the heart and kidney models do not rely on this feature it is understandable that liver_kidney and liver_heart cross-tissue models show an unexpected decrease in performance when DNA shape and histone modification features are added to the sequence features. On the other hand, kidney_liver and heart_liver cross tissue models show a boost in performance with the addition of histone modification features. These results suggest that there is some degree of commonality in BMAL1 binding between different tissues. However, in cross-tissue models sequence-only models exhibit the most robust performance with the exception of the kidney_heart and heart_kidney models, indicating that DNA shape and chromatin context features can exhibit high degree of tissue specificity and are more similar between kidney and heart than they are between liver and the other two tissues.

E-box binding specificity of the yeast bHLH TFs Cbf1 and Tye7 is governed by sequences flanking the E-box as reflected in DNA shape^54^. Our findings extend this concept, indicating that not only might DNA shape and chromatin context confer different binding specificities to different TFs in the same tissue, but that they might also confer different binding specificities to the same TF in different tissues.

## Supplementary Information

Supplementary Information 1.

Supplementary Information 2.

Supplementary Information 3.

Supplementary Information 4.

Supplementary Information 5.

Supplementary Information 6.

Supplementary Figures.

Supplementary Information 7.

## Data Availability

The ChIP-seq datasets used for this study are available in GEO (accession number GSE110604) from a previous study^9^. DNase-seq and Histone modification datasets accession numbers are included in the supplementary material. The code used in the machine learning modeling are available at https://github.com/BhattacharyaLab.

## References

[1] Ko CH, Takahashi JS (2006). Molecular components of the mammalian circadian clock. Hum. Mol. Genet..

[2] Takahashi JS, Hong HK, Ko CH, McDearmon EL (2008). The genetics of mammalian circadian order and disorder: implications for physiology and disease. Nat. Rev. Genet..

[3] Landgraf D, Wang LL, Diemer T, Welsh DK (2016). NPAS2 compensates for loss of CLOCK in peripheral circadian oscillators. PLoS Genet..

[4] Cox KH, Takahashi JS (2019). Circadian clock genes and the transcriptional architecture of the clock mechanism. J. Mol. Endocrinol..

[5] Yoo SH (2005). A noncanonical E-box enhancer drives mouse Period2 circadian oscillations in vivo. Proc. Natl. Acad. Sci. USA.

[6] Kathiresan S, Srivastava D (2012). Genetics of human cardiovascular disease. Cell.

[7] Schödel J (2012). Common genetic variants at the 11q13.3 renal cancer susceptibility locus influence binding of HIF to an enhancer of cyclin D1 expression. Nat. Genet..

[8] Lambert SA (2018). The human transcription factors. Cell.

[9] Beytebiere JR (2019). Tissue-specific BMAL1 cistromes reveal that rhythmic transcription is associated with rhythmic enhancer–enhancer interactions. Genes Dev..

[10] Dror I, Golan T, Levy C, Rohs R, Mandel-Gutfreund Y (2015). A widespread role of the motif environment in transcription factor binding across diverse protein families. Genome Res..

[11] Morgunova E, Taipale J (2017). Structural perspective of cooperative transcription factor binding. Curr. Opin. Struct. Biol..

[12] Wang J (2012). Sequence features and chromatin structure around the genomic regions bound by 119 human transcription factors. Genome Res..

[13] Zhou T (2015). Quantitative modeling of transcription factor binding specificities using DNA shape. Proc. Natl. Acad. Sci. USA.

[14] Filipovic, D. *et al.**Predictive Models of Genome-Wide Aryl Hydrocarbon Receptor DNA Binding Reveal Tissue Specific Binding Determinants*. *bioRxiv* 2022.05.13.491754. 10.1101/2022.05.13.491754 (2022). 10.1093/toxsci/kfad094 PMC10682972 37707797

[15] Steuernagel L (2019). Computational identification of tissue-specific transcription factor cooperation in ten cattle tissues. PLoS ONE.

[16] Barski A (2007). High-resolution profiling of histone methylations in the human genome. Cell.

[17] Arvey A, Agius P, Noble WS, Leslie C (2012). Sequence and chromatin determinants of cell-type-specific transcription factor binding. Genome Res..

[18] Gordân R (2013). Genomic regions flanking E-box binding sites influence DNA binding specificity of bHLH transcription factors through DNA shape. Cell Rep..

[19] Pique-Regi R (2011). Accurate inference of transcription factor binding from DNA sequence and chromatin accessibility data. Genome Res..

[20] Das MK, Dai HK (2007). A survey of DNA motif finding algorithms. BMC Bioinform..

[21] Alipanahi B, Delong A, Weirauch MT, Frey BJ (2015). Predicting the sequence specificities of DNA- and RNA-binding proteins by deep learning. Nat. Biotechnol..

[22] Quang D, Xie X (2016). DanQ: A hybrid convolutional and recurrent deep neural network for quantifying the function of DNA sequences. Nucleic Acids Res..

[23] Zhou J, Troyanskaya OG (2015). Predicting effects of noncoding variants with deep learning-based sequence model. Nat. Methods.

[24] Chen, T. & Guestrin, C. XGBoost: A scalable tree boosting system. in *Proceedings of the ACM SIGKDD International Conference on Knowledge Discovery and Data Mining*, 13–17-August-2016. 785–794 (2016).

[25] Mathelier A (2016). DNA shape features improve transcription factor binding site predictions in vivo. Cell Syst..

[26] Wang Y, Li X, Hu H (2014). H3K4me2 reliably defines transcription factor binding regions in different cells. Genomics.

[27] Slattery M (2014). Absence of a simple code: How transcription factors read the genome. Trends Biochem. Sci..

[28] Li J (2017). Expanding the repertoire of DNA shape features for genome-scale studies of transcription factor binding. Nucleic Acids Res..

[29] Quinlan AR, Hall IM (2010). BEDTools: A flexible suite of utilities for comparing genomic features. Bioinformatics.

[30] Chiu TP (2016). DNAshapeR: An R/Bioconductor package for DNA shape prediction and feature encoding. Bioinformatics.

[31] Ramírez F (2016). deepTools2: A next generation web server for deep-sequencing data analysis. Nucleic Acids Res..

[32] Pohl A, Beato M (2014). bwtool: A tool for bigWig files. Bioinformatics.

[33] Peng CYJ, Lee KL, Ingersoll GM (2010). An introduction to logistic regression analysis and reporting. J. Educ. Res..

[34] Wang Z, Wu Y, Li L, Su XD (2012). Intermolecular recognition revealed by the complex structure of human CLOCK–BMAL1 basic helix–loop–helix domains with E-box DNA. Cell Res..

[35] Liu S (2017). Assessing the model transferability for prediction of transcription factor binding sites based on chromatin accessibility. BMC Bioinform..

[36] Untitled Diagram—diagrams.net. https://app.diagrams.net/?src=about.

[37] Zhou T (2013). DNAshape: A method for the high-throughput prediction of DNA structural features on a genomic scale. Nucleic Acids Res..

[38] Benveniste D, Sonntag HJ, Sanguinetti G, Sproul D (2014). Transcription factor binding predicts histone modifications in human cell lines. Proc. Natl. Acad. Sci. USA.

[39] Guccione E (2006). Myc-binding-site recognition in the human genome is determined by chromatin context. Nat. Cell Biol..

[40] Xin B, Rohs R (2018). Relationship between histone modifications and transcription factor binding is protein family specific. Genome Res..

[41] Heintzman ND (2009). Histone modifications at human enhancers reflect global cell-type-specific gene expression. Nature.

[42] Ramsey SA (2010). Genome-wide histone acetylation data improve prediction of mammalian transcription factor binding sites. Bioinformatics.

[43] Korobov, M. & Lopuhin, K. *ELI5 Documentation Release 0.11.0*. (2021).

[44] Crooks GE, Hon G, Chandonia JM, Brenner SE (2004). WebLogo: A sequence logo generator. Genome Res..

[45] Levine M, Tjian R (2003). Transcription regulation and animal diversity. Nature.

[46] Mure LS (2018). Diurnal transcriptome atlas of a primate across major neural and peripheral tissues. Science.

[47] Hogenesch JB, Gu YZ, Jain S, Bradfield CA (1998). The basic-helix–loop–helix-PAS orphan MOP3 forms transcriptionally active complexes with circadian and hypoxia factors. Proc. Natl. Acad. Sci. USA.

[48] Zhang R, Lahens NF, Ballance HI, Hughes ME, Hogenesch JB (2014). A circadian gene expression atlas in mammals: Implications for biology and medicine. Proc. Natl. Acad. Sci. USA.

[49] Shimomura K (2013). Usf1, a suppressor of the circadian Clock mutant, reveals the nature of the DNA-binding of the CLOCK:BMAL1 complex in mice. Elife.

[50] Menet JS, Pescatore S, Rosbash M (2014). CLOCK:BMAL1 is a pioneer-like transcription factor. Genes Dev..

[51] Wang S (2021). Predicting transcription factor binding sites using DNA shape features based on shared hybrid deep learning architecture. Mol. Ther. Nucleic Acids.

[52] Gupta P, Zlatanova J, Tomschik M (2009). Nucleosome assembly depends on the torsion in the DNA molecule: A magnetic tweezers study. Biophys. J..

[53] Koike N (2012). Transcriptional architecture and chromatin landscape of the core circadian clock in mammals. Science.

[54] Grove CA (2009). A multiparameter network reveals extensive divergence between *C. elegans* bHLH transcription factors. Cell.

